# Coupling Plant Growth Models and Pest and Disease Models: An Interaction Structure Proposal, MIMIC

**DOI:** 10.34133/plantphenomics.0077

**Published:** 2023-08-04

**Authors:** Houssem E. M. Triki, Fabienne Ribeyre, Fabrice Pinard, Marc Jaeger

**Affiliations:** ^1^ CIRAD, UMR AMAP, F-34398 Montpellier, France.; ^2^ AMAP, University of Montpellier, CIRAD, CNRS, INRAE, IRD, Montpellier, France.; ^3^ CIRAD, UMR PHIM, F-34398 Montpellier, France.; ^4^ PHIM, University of Montpellier, CIRAD, INRAE, Institut Agro, IRD, Montpellier, France.; ^5^ CIRAD, UMR PHIM, 00100 Nairobi, Kenya.

## Abstract

Coupling plant growth model with pests and diseases (P&D) models, with consideration for the long-term feedback that occurs after the interaction, is still a challenging task nowadays. While a number of studies have examined various methodologies, none of them provides a generic frame able to host existing models and their codes without updating deeply their architecture. We developed MIMIC (Mediation Interface for Model Inner Coupling), an open-access framework/tool for this objective. MIMIC allows to couple plant growth and P&D models in a variety of ways. Users can experiment with various interaction configurations, ranging from a weak coupling that is mediated by the direct exchange of inputs and outputs between models to an advanced coupling that utilizes a third-party tool if the models’ data or operating cycles do not align. The users decide how the interactions operate, and the platform offers powerful tools to design key features of the interactions, mobilizing metaprogramming techniques. The proposed framework is demonstrated, implementing coffee berry borers’ attacks on *Coffea arabica* fruits. Observations conducted in a field in Sumatra (Indonesia) assess the coupled interaction model. Finally, we highlight the user-centric implementation characteristics of MIMIC, as a practical and convenient tool that requires minimal coding knowledge to use.

## Introduction

Agroecological transition is an active research and development area, in which modeling agronomical system productions must be assessed from complex systems modeling in regard to the processes involved in and their interactions. As mentioned by Brandmeyer and Karimi [1], “complex environmental problems involve processes that occur both within and between environmental media”; thus, both aspects must be considered to build efficient model couplings.

The authors underline the difficulties in handling how the different models interact with each other. In each field of study, modeling communities developed their own techniques and frameworks for creating efficient simulation models of the processes they are interested in [2]. Nevertheless, certain problems and complications are not specific to one discipline.

In particular, the integration of the potential intertwined effects that the dynamics of certain models may have on each other and the compatibility of their architecture [3] focus our interest.

Indeed, we are interested in an application in the environmental domain, more specifically, in estimating plant production under certain conditions. In general, the productivity of a plant that has been attacked by a pest or a disease (pest and disease (P&D)) is assessed in the short term. P&D directly damages one or more organs of the plant by targeting them. If the infected organs encompass the fruits, then yield reductions are direct. Production projections at mid- and long term are seldom considered.

**Fig. 1.**
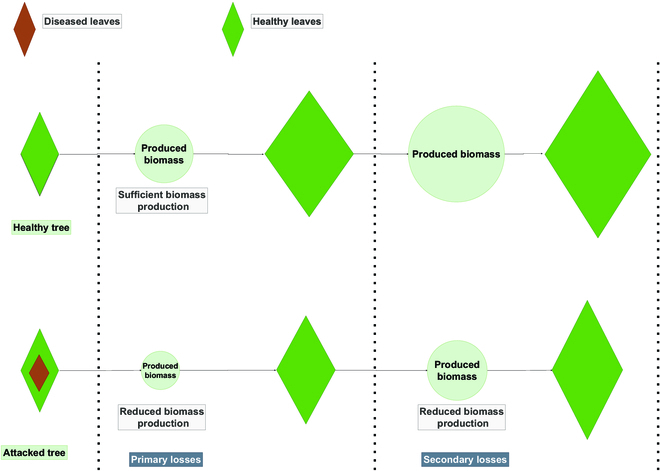
Interactions between P&D dynamics and coffee phenology. Case of a leaf disease affecting the biomass accumulation with a cumulative effect on the long-term plant growth.

However, even if the fruits are not directly affected by the attack, the future yield and growth of the plant are usually affected because of the decrease in biomass production (e.g., an attack on the leaves reduces light interception). In addition, these effects alter plant growth by changing the balance between organs and potentially the distribution of resources; Fig. 1 illustrates such an effect.

**Table 1.** CBB’s cohort distribution based on population hatching day and colonized fruit category.

CBB population/fruit category	*Hatched the 1st day*	*Hatched the 2nd day*	*…*	*Hatched the nth day*
*Very Appealing fruits*	Cohort (1, VAp)	Cohort (2, VAp)	…	Cohort (n, VAp)
*Appealing fruits*	Cohort (1, Ap)	Cohort (2, Ap)	…	Cohort (n, Ap)
*Fruits on the grounds*	Cohort (1, G)	Cohort (2, G)	…	Cohort (n, G)

These feedback effects are rarely taken into account when coupling descriptive models. This is, in part, due to the lack of mechanistic dynamic approaches reported at the organ level to model and simulate the interactions between plant growth and P&D attacks [4]. Therefore, feedback to the plant itself can hardly be assessed. Another critical point is that, even when mechanistic models are available, their coupling potential is not evaluated, both for computational cycles and for the data on which they interact with each other.

To determine more precisely the thresholds at which P&Ds are likely to have a important economic impact on production [5] and to make an appropriate treatment decision, a coupled model operating over the long term would be helpful.

Such a model could provide a more accurate estimation of the effects of climate change on plant phenology/biotic development. In the last decade, Uganda has experienced a great resurgence of P&Ds in coffee tree fields [6,7] despite the important efforts of national institutes to create resistant clones, especially for coffee wilt disease (*Fusarium xylarioides*). In addition to this, climate change induces new environmental conditions affecting plant growth dynamics and the dynamics of pathogens and insects [8–10]. Consequently, the coffee berry borer (CBB) (*Hypothenemus hampei*) and coffee leaf rust (CLR) (*Hemileia vastatrix*) are present in Uganda, although at moderate levels. However, severe cases of red blister (*Cercospora coffeicola*), which affects leaves and berries; of black twig borer (*Xylosandrus compactus*) [11]; and of coffee wilt disease [12–14] are now reported. This region combines multiple P&Ds that affect coffee trees at various organs and scales. Thus, we consider it as a good experimental field for an application of coupling framework.

### Objectives and scope

Our overall goal is to assess variation in production linked to P&D and related mechanisms at the plant and crop scales, as well as the impact of crop practices. These elements can only be accessed through models. In other words, as suggested by Cerda et al. [15] and Gaunt [16], the estimated effects of P&D on plants are considered to result from interactions between dynamic models: the plant growth model (considered at least at the organ scale), the P&D model, and even a human intervention model (treatment, harvest, etc.). We are thus facing a complex system involving processes that interact at different levels, with the possibility of collective behaviors and relationships with the environment [17].

We propose here a specific coupling framework taking into account (a) the specific difficulties encountered when dealing with complex systems (cycle synchronization, data concurrency and sharing, etc.); (b) the specific difficulties related to the nature and type of models involved for plant growth and P&D dynamics models; (c) the limitation of technical complexity, as users are usually not specialized in automation and their convex domains; and (d) the possibility to add models without modifying other models already involved in the framework.

### About plant growth models

In agronomy, process-based models (PBMs) and functional structural plant models (FSPMs) are 2 major frameworks to model plant growth dynamics.

PBMs, generally referred to as crop models, estimate the biomass produced mainly from the intercepted light by leavesper unit area (m^2^)[18]. The different organs are grouped into distinct compartments. The distribution of produced biomass within each compartment is differentiated, allowing yield estimation. However, PBMs do not consider the organ typology resulting from the plant’s structure, the plant phenology, nor the mechanisms that occur within the plant.

The FSPM aims at modeling the establishment of the plant structure and the functioning of the organs. In FSPM, the properties and functions of each organ are taken into account [19]. In particular, the distribution of biomass produced by the different organs is followed at the scale of each organ in the whole plant. Such models operate on the individual plant scale and generate an explicit 3D geometric representation of the plant. However, using FSPM formalism requires calibrations of the model parameters that are often cumbersome.

Nevertheless, with a limited number of assumptions and the use of certain PBM formalisms, it is possible to factorize the processes involved in FSPM, based on the attributes of the organ structural criterion, and generate a model with a reduced complexity [20]. Such a design is illustrated by the GreenLab formalism [21]. It applies the notion of a common biomass pool, assessed on several crops of interest [22]; uses the traditional light interception model of PBM; and operates with cohorts of organs defined from chronological and physiological ages [23,24]. Furthermore, this style of formalism allows to move from the individual plant level to the crop level.

### About P&D models

There are numerous ways to design and qualify P&D models. We distinguish here 2 categories, statistical models and mechanistic models.

Statistical models are developed from data and statistical correlations between model variables. These models are capable of prediction but are difficult to transfer; they are difficult to project beyond the spatial and temporal boundaries of their underlying data [25].

Mechanistic P&D models include explicit hypothesis on biological mechanisms that influence the dynamics of the P&Ds. These models can be used to simulate the P&Ds on different scales (plots, regions, countries, etc.) [26,27]. Notions of cohorts are also often inherent in these approaches, quantifying populations of similar age and behavior.

### Plant and P&D model coupling

Conceptually, coupling 2 or more processes together falls into one of the following categories, inspired by [28]:

1. Sequential coupling or loose coupling: Models are completely decoupled or models exchange data through inputs/outputs (I/O).
2. Shared coupling: (a) GUI (graphical user interface): Models share a common GUI or (b) Data: Models share the full I/O database.
3. Embedded or integrated: One model is fully contained within the other (usually as a subroutine) or model codes are merged into a single coherent model.
4. Framework coupling: Use of a global modeling framework, where the models are coupled using a third-party tool commonly called “Coupler,” based on a combination of the previous methods.

The literature on coupling plant and P&D models to estimate plant production agrees that this is a challenge, requiring more physiological and field studies [29]. Studies are still scarce on the subject today. The most popular categories of coupling are loose coupling and integrated coupling.

The DynACof coffee plant model [30] is a dedicated PBM. In a recent study, it was linked to a rust model, with a loose coupling defined by the ratio of healthy to damaged leaf area [31].

Interaction through data is easy to manage, but this type of coupling requires some synergy between models. It is difficult to generalize to multiple models or interaction cases if the I/O do not match the requirements of each model. To circumvent this type of problem, a modeling environment may be mobilized such as OpenAlea [32]. This platform provides users an interface to create interactions between plant (sub)models and their environments. OpenAlea allows couplings ranging from simple sequential to shared coupling; users can simply define model sequences or build graphs to connect I/O models with a generic graphical interface: VisuAlea. The platform also provides multiple analysis and visualization tools. However, even if users are able to produce interacting models, this still requires coding to adjust or add other components [33].

The theoretical study conducted by Qi et al. [34] models the palm tree under pest attack, whose population is also constrained by auxiliary insects; the population dynamics and attack models of P&D integrate the plant growth model. This is a case of embedded and integrated coupling. Here, the feedback on biomass and plant populations is well evaluated over the long term, but while the plant model is generic, the insect models and interactions are not: Attacks are limited to leaf damage, and synchronization is implicit and climate conditions are supposed to be stable.

Recently, Motisi et al. [35] propose an integrated approach to such a system by decomposing the P&D model (leaf rust) and the growth model (coffee) into smaller models, acting as submodels embedded in each other, building an integrated coupling.

Le Chevalier et al. [36] developed a framework, inspired by the DEVS (Discrete Event System Specification) mathematical formalism [37], involving a simple big leaf implementation of the GreenLab model with a climate model (rainfall and temperature) and a water diffusion model (runoff, soil diffusion, and plant uptake); this framework allows modeling growth variability related to plant competition for water and local conditions such as altitude and orientation.

A common drawback of these examples is their lack of generality, especially regarding adding/changing P&D models or changing plant species. This is reflected in the limited literature that combines abiotic (climate) and biotic (P&D and/or farmer) influences on the plant [4]. Classically, model interactions are assessed by mutually coupling models together. However, this method is case specific and difficult to generalize even using a platform such as OpenAlea, as highlighted by Garin et al. [38].

The work presented here is part of a study focusing on P&D attacks on Robusta coffee in Uganda and, more specifically, on fruits with red blister and CBB; on leaves with CLR; and on young branches with black twig borer. We address modeling the interactions occurring at various levels of the plant with different P&D models over a large period (theoretically, the entire life span of the plant). To this end, our proposal is based on a “framework coupling” that encompasses the various coupling categories outlined previously [28].

In the following contribution, the rationales and components of the proposed model coupling framework are presented first. The framework’s architecture and implementation are then explained. We then present a case study including the CBB and a fructification model. Before concluding, the framework structure genericity and assumptions are discussed.

## Materials and Methods

### Framework design assumptions

Although our applications are dedicated to the evaluation of coffee production under P&D attacks, we aim to develop a generic approach, adapted to many interactions mobilizing agronomical models, but not as generic as an implementation under DEVS (some arguments are given in the discussion) [37]. The latter would strongly put constrains on how to define the models and their inner mechanisms, particularly with regard to their synchronization procedure.

OpenMole could also be considered as an alternative [39]. However, OpenMole is not strictly a model coupling environment, and it does not provide an environment to design models from scratch. OpenMole shows high interest for analyzing existing interaction codes. It helps modelers to evaluate the sensitivity of their models’ parameters and optimize them. Once finalized, at exploitation stage, OpenMole offers valuable upscaling deployment mobilizing cloud or HPC.

In MIMIC (Mediation Interface for Model Inner Coupling), our focus is to assist the user in the creation of the basic structures of the interaction. We may thus consider that an interaction model could be first created with MIMIC and then be integrated into management environments such as OpenMole.

Before describing our approach, we list here the founding assumptions of our framework.

Because we aim to integrate feedback on the plant growth model, we focus on developing the framework at the individual plant scale. This self-imposed condition allows us to implicit the spatial aspect involved in many P&D attacks.

Associating the effects of the dynamics of different models can be complicated, especially when considering models that work on different time scales or natures (chronological or thermal) and when considering feedback. The users must therefore be able to define a cycle correspondence between the third party and each model.

Among the different formalisms of plant growth models mentioned above, the adoption of a cohort-based formalism allows flexibility in the coupling of models by reducing the number of parameters required for calibration [40]. In addition, this formalism offers the advantage of incorporating plant growth feedback on organogenesis for the expression of plasticity in a competitive context [41] and provides practical means for parameter evaluation [42], including the case of functional feedback on the plant structure [43]. The structure calculation is implicit; calculations are factorized as defined by the number of organ types and cohorts (organ ages and physiological states), leading to short computation times [44] and, thus, minimizing interaction complexity. For P&D models, some correspondence with the type of plant model is necessary to reduce the complexity of the coupled system. For this reason, models using groups (cohorts) of populations are preferred.

Some P&D models are “individual-based” or based on age groups to take into consideration that various stages of development do not always react the same way to environmental factors (sometimes called cohort). In study of Rodríguez et al. [45], the term “cohort” designs a group of plants and CBB that are all the same age.

This assumption allows to take cohorts from each model and to create new ones with flexible criteria (see the discussion first section). Thus, the users can manipulate the outputs of the models and even add new interaction-related variables to define the new cohorts.

The proposed approach is framework based, which means that all models involved interact through a third party and do not interact directly with each other. The models involved must be able to initiate a computation at a given step, reading their inputs and providing outputs for the requested step. The models mobilized to interact should not be heavily modified, and their internal structure or operation must not be altered by the coupling.

### MIMIC, a formal framework for interaction

In this study, the third-party tool is called MIMIC. MIMIC handles interactions between models in a flexible way, regardless of the number and types of models. In addition, while the development is based on the interplay between P&D and the plant growth model, this framework is not exclusive to P&D. MIMIC ensures coupling effects on the dynamics of all models on a long-term time scale.

#### MIMIC: Overview and principles

MIMIC manages the connection between models and their inputs and outputs. The fundamental assumption of the model is that each model operates and evaluates its own internal states in a finite amount of time, from one internal step to the next. MIMIC supervises the interaction through its own states and information, which are evaluated on the basis of the information collected from the connected models.

On the basis of the information obtained from the output of the models, MIMIC manages the underlying mechanics of the interaction between the components; there is no limit to the number of models that can be linked together. This reduces the expected complexity of such system combinations and allows for easy handling and independence of the models. Moreover, when developing a new application (adding elements and changing dynamics), no changes are required to MIMIC’s kernel. This is an advantage from a development point of view because each component execution process is distinct and can be modified independently, which sustains the correct execution of the framework.

#### MIMIC: The components

**Fig. 2.**
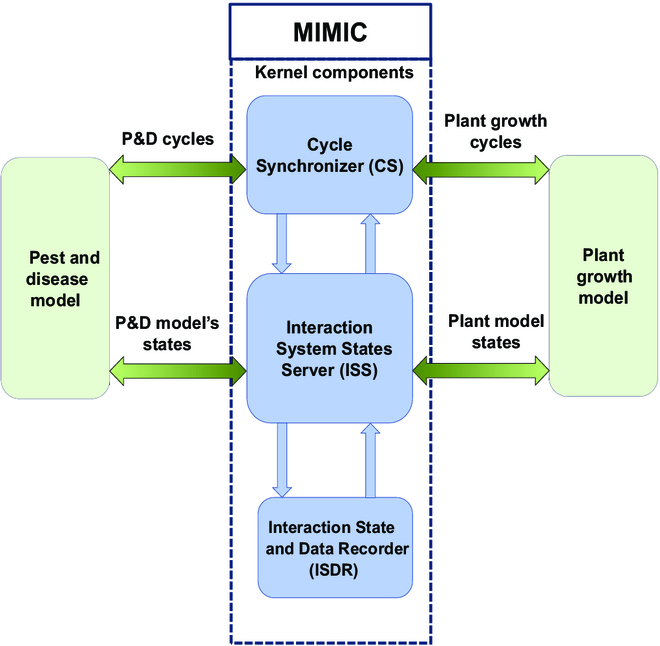
The “Mediation Interface for Model Inner Coupling” (MIMIC) schematics, illustrating the kernel components (in blue) on a coupling example with a P&D model (left green box) and a plant growth model (right green box).

MIMIC can be considered as a hyper model consisting of 3 main components, as shown in Fig. 2 in its central part. These components solve the problems classically encountered when coupling different and multiple dynamic systems, from desynchronization to feedback integration.

The first component schedules the simulations according to the temporalities of the connected models. The Cycle Synchronizer (CS) executes the connected models and starts the interaction process between the involved models. This component prioritizes multiple interacting models based on users’ preferences. The second component, the Interaction System States Server (ISS), serves the communication protocols used to create the MIMIC state variables (variables used for interactions). Finally, the third component, the Interaction State and Data Recorder (ISDR), ensures the integrity of the interaction and the state variables of the coupling interface connecting the interacting models. It also manages data and other dynamics that are not required for interaction but can be requested by the users for observation purposes.

Cycle Synchronizer (CS)

**Fig. 3.**
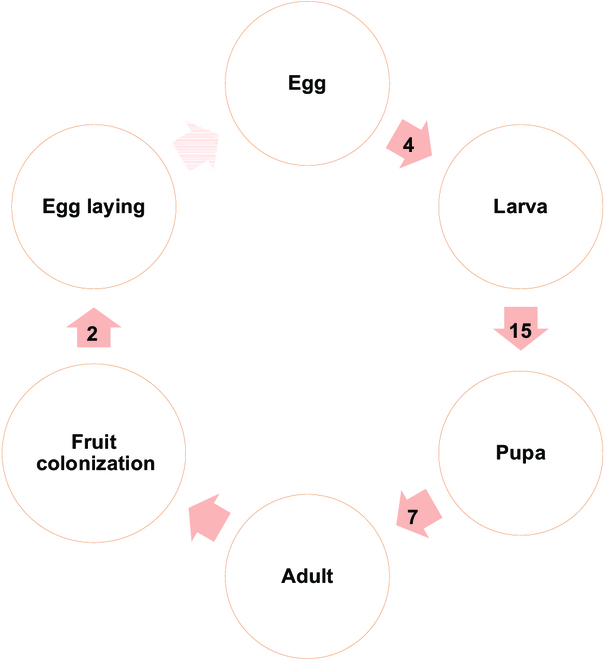
Female CBB life cycle representation. Circles stand for the stages. Arrows stand for transitions with their average duration in days. Light-colored arrow means the restart of the cycle for another generation.

The CS component is built around a specific behavior design pattern called Mediator (Fig. 3), which describes how objects interact with each other. The Mediator promotes loose coupling by preventing objects from referring to each other explicitly and allows some independence between them [46]. A so-called “behavioral model” reduces chaotic dependencies between components. It forbids direct communication between them and requires them to collaborate only through the Mediator.

The activities of each model and component are scheduled using this Mediator-based component. Thus, it addresses the problems related to desynchronization. An essential point concerns the definition of scheduling. Typically, as defined in the DEVS formalism [47], a temporal base reference must be addressed to each connected model, and each model must be able to return its own cycle conversion step in the temporal reference.

For example, many P&D models operate by generations resulting from climate data (and other parameters), but plant models typically define their cycle from organogenesis (from a thermal calendar). In this case, an effective method is to have the users set the default time cycle in the interface based on the smallest cycle of the models available during the initialization phase and then mobilize a function (e.g., a function related to climate data) to retrieve the average daily temperature from which the interface calculates the date of the next plant growth stage.

Interaction System States Server (ISS)

When one of the models is active (running), its state variables pass through this component and are converted to MIMIC’s state variables. This operation creates a state variable that can be understood by any other model involved in the interaction. The definition and translation of the ISS state variables are the responsibility of the users, based on their knowledge of the interacting models and the desired observations. State variables are of 3 types: (a) state variables considered as output copies of the connected models, (b) state variables specific to the internal operations of MIMIC, and (c) state variables defined by the users to encode the interaction between models and MIMIC.

Interaction State and Data Recorder (ISDR)

In this component, in addition to interaction-relevant states, data collected from connected models and internal data resulting from computations within the interface are recorded and stored at each event (when any model is executed) processed in the schedule. The storage of state variables and data from the coupling model interface makes stop-and-go simulation available. In a broader sense, storing interaction state and data allows users to access a simulation from a previous date in the scheduler, rerun it, and simulate different scenarios by dynamically adjusting the simulation parameters. The results of the interaction simulation are accessible directly from MIMIC, without going through the associated models. The results are adaptable, independent of the simulation itself, and can be read backward and forward (to the last event of the simulation).

To illustrate the MIMIC framework, a simple attack case is considered below.

### A case study: The CBB and Arabica coffee

In this simple example, we consider the CBB that attacks coffee berries. We assume that, at the time scale considered, there is no visible effect on plant growth.

#### The CBB model

*H. hampei* (Ferrari) is a pest known as CBB, belonging to the order Coleoptera, family Curculionidae, and subfamily Scolytinae [48]. This small beetle originates from Central Africa and is present in all coffee-producing countries of the world.

A CBB hatches from an egg in the seed of a coffee berry. When the fertilized females leave the fruit, they colonize another one and start their own colony (Fig. 3). The factors triggering the exit from the fruit are the age of the insect and the climate (temperature, humidity, and rain). CBB is attracted to by red (ripe) fruits and green fruits (if they are large enough). The insect is more attracted by red than green fruits; however, if the number of attractive fruits is small, then CBB will colonize overripe fruits or fruits that have fallen to the ground. The average life span of a female CBB is nearby 45 d [49].

Fruits are grouped into 3 distinct categories. “Very attractive fruits” (VAp) are ripe fruits, from the moment they turn red. “Attractive fruits” (Ap) are well-developed green fruits. This category includes green fruits larger than 5 mm, with seeds capable of hosting the CBB, to fruits that turn yellow. “Ground fruits” (G) are all fruits fallen to the ground, whatever the former category they belonged to. In the proposed model, each of the above fruit categories has an attraction factor that influences whether CBB chooses to colonize a fruit or not.

Population monitoring is based on the grouping of different individuals within a population having the same oviposition day. Male CBBs are not considered in monitoring of populations as they do not play any role in the epidemic propagation (they represent only 110 of the individuals in a colony and are not disseminated). As already mentioned, population dynamics depend on temperature, relative humidity, and precipitation.

The cohorts of the model are built by crossing the groups of individuals and the categories of fruits where these individuals live.

On each simulation step, the results of new attacks are grouped into 2 categories: population data and fruit data. The population data contain information about each population group for a given day. It contains the date when this group left its original fruit to colonize other fruits, the number of flying CBB, the number of dead CBB, and so on. The fruit data include the fruit categories presented above. In addition, they are divided into 2 subgroups, healthy and colonized fruit. The result is a cohort of fruit categories attacked by a quantity of CBB hatched on a given day.

#### The coffee fruit cohort model

In the Sumatra region of Indonesia, Arabica coffee trees produce coffee berries throughout the year. With the presence of rainfall throughout the year and an average daytime temperature between 22 and 30 °C, the equatorial climate provides the necessary conditions for the trees to flower. A plant growth model is created as a reduced model to simulate fruiting only. We designed a cohort fruit model inspired by the GreenLab cohort assumption: Fruits with the same parameters (chronological age, physiological age, and sink power) are merged into the same cohort. An automaton is created that build fruit cohorts on the basis of the obseved numbers of berries harvested. Then, the model estimates the age of the fruits according to the harvest frequencies and the climatic data.

#### Human intervention model

Human intervention is represented here as a simple harvest model. This model simulates harvesting of red berries at dates that correspond to observed data, which is useful for validation by comparing simulated data to actual data.

#### Integration of the coffee–CBB interaction

When CBB attacks a fruit on the tree, the inner seed is damaged, but the fruit continues to develop and the biomass is still distributed. We therefore consider that the feedback on the plant is negligible. Thus, in this case, the interaction focuses on the state of the fruits (attacked or healthy).

Interaction between the 2 models is achieved by converting the numbers of attacked fruits provided by the CBB model into the cohorts of the plant model An additional state variable ISS is created in MIMIC: the status of the cohort. This additional data is Boolean, indicating whether the cohort is colonized by the CBB.

#### Validation data

To validate the functioning of the interface, we used climatic, fruiting, and attack data on 2 coffee trees in Indonesia for almost a year [50]. The observations were not made daily but separated by slightly irregular periods of time (about 20 d between each observation). This implies a daily operation of the model and an estimated chronological age of the initial populations. The solution chosen was to consider maturation occured exactly between 2 observations. A CBB colony is established at the start of the simulation and begins its development. The number of colonized fruits is initialized by the observed data. Because there is no data for fruit on the ground, this category was discarded from the simulation.

The study case and simulation results are presented below, after detailing the overall implementation aspects.

## Implementation and Results

We present here the implementation of MIMIC, starting with the architecture before detailing the components and some specific features and functions.

### Architecture (kernel, pseudo-models, and models) of MIMIC

**Fig. 4.**
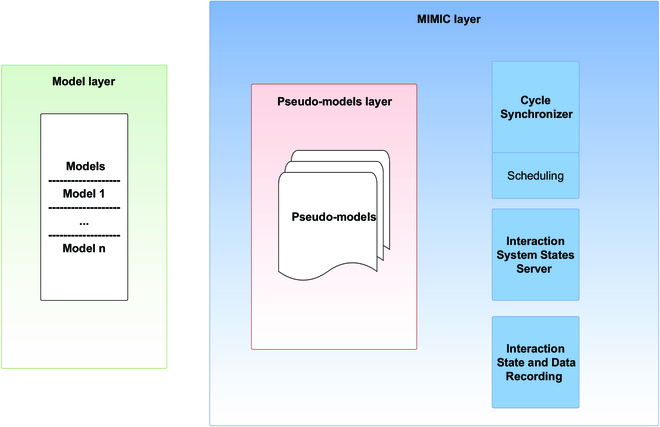
MIMIC’s and models’ layer composition. The framework is composed of 3 layers: layer 1 (in green) stands for the external models, layer 2 bloc (in red) contains the pseudo-models, and layer 3 (in blue) contains MIMIC’s functioning components.

Using MIMIC, to integrate models into an interaction structure, results in a software architecture composed of 3 layers (Fig. 4). The first layer consists of the models involved in the coupling. These models are independent and external to MIMIC. They are only linked to MIMIC through the second layer: the pseudo-model’s layer.

**Fig. 5.**
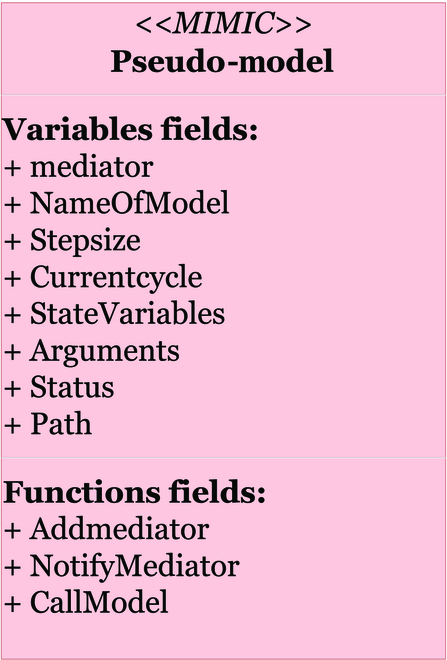
Representation of pseudo-models as Unified Modeling Language (UML) (red bloc in Fig. 4). The structure of a pseudo-model (generated by MIMIC) with its variable and function fields. Mediator fields link the pseudo-model with the kernel, while the “CallModel” function links to its “external model.” Note that in this UML diagram (and the following one), variables are values (input/output) used by the component, while functions are specific programs related to the component.

**Fig. 6.**
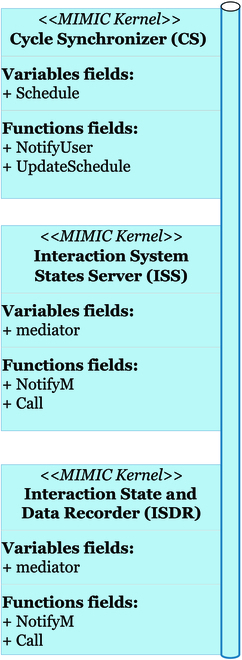
MIMIC’s kernel UML representation (blue bloc in Fig. 4), describing the data structure of MIMIC’s main components (Cycle Synchronizer, Interaction State and Data Recorder, and Interaction System States Server) linked through a communication bus (blue cylinder).

The pseudo-models (red blocs in Figs. 4 and 5) are wrappers of the interacting models of the first layer. This association is unique and bijective. This layer is automatically created by MIMIC at the initialization stage. The pseudo-models provide all the information about the models, which are requested at run time. The third layer (blue blocs in Figs. 4 and 6) is the core of the MIMIC protocol. Composed of 3 components, it schedules, manages tasks, and manages data exchanges. The 3 specific components are described below.

### Functioning of MIMIC’s components

#### Models’ layer

Models are independent of MIMIC. They exchange only data with MIMIC (state variables, cycles, etc.) each time an execution call is processed.

#### Pseudo-models’ layer

Pseudo-models are automatically generated by MIMIC in an initialization process, from parameters and information related to the models, provided by the user (Fig. 5) filling a YAML file (see the User layer here under) [51]. The generated pseudo-model’s variables host the data requested for simulation and interaction (state variables, arguments, step size, and path to the model code), as well as the current model cycle and state (running, pending, or unsolicited). The “mediator” field links the pseudo-model to the kernel.

MIMIC also generates the pseudo-models’ functions. “Call model” refers to the function used to call the related model. The 2 other functions are the constructor of the link to the MIMIC kernel (using “Addmediator”) and the constructor of the notifications to the kernel (using “Notifymediator”).

#### MIMIC’s kernel layer

The 3 components of the MIMIC’s kernel (CS, ISS, and ISDR) are directly connected to one another through a shared communication bus (Fig. 6).

The CS controls how MIMIC manages and runs processes (Fig. 6, top). The function “UpdatesSchedule” is called each time a model or component is requested to close an event; it updates the list of scheduled tasks (Table 2), stored in the variable “Schedule.” This type of dynamic scheduling allows interaction between models with different and varying step times or time natures, e.g., calendar and thermal time.

**Table 2.** The case study tasks list and the schedule after 10 simulation steps. (A) List of events submitted to the scheduler. (B) The events agenda of the scheduled tasks. On both lists, parameters are the following, from left to right: name of model (Model3 stands here for the human model), step size for the model, last time executed, next time to be executed, and status in the schedule.

A.Submitted event list					
Rank	Process Id (name)	Step size	Order time	Exec time	Status
1	Tasks_Desk «CBB»	3.0	0.0	3.0	«Completed»
2	Tasks_Desk «Coffee_tree»	4.0	0.0	4.0	«Completed»
3	Tasks_Desk «CBB»	3.0	3.0	6.0	«Completed»
4	Tasks_Desk «Model3»	8.0	0.0	8.0	«Completed»
5	Tasks_Desk «Coffee_tree»	4.0	4.0	8.0	«Completed»
6	Tasks_Desk «CBB»	3.0	6.0	9.0	«Completed»
7	Tasks_Desk «Coffee_tree»	4.0	8.0	12.0	«In Queue»
8	Tasks_Desk «Model3»	8.0	8.0	16.0	«In Queue»
9	Tasks_Desk «CBB»	3.0	9.0	12.0	«In Queue»

B.Tasks scheduled at time 10					
1	Tasks_Desk «Coffee_tree»	4.0	8.0	12.0	«In Queue»
2	Tasks_Desk «CBB»	3.0	9.0	12.0	«In Queue»
3	Tasks_Desk «Model3»	8.0	8.0	16.0	«In Queue»

At each event, the “NotifyUser” function send messages to the console in a log file, allowing the user to follow the simulation step by step.

The ISDR stores interaction-relevant state variables and simulation data (task schedule, last model run, etc.) step by step. This simulation data logging is convenient for stop-and-go implementation. The ISDR is also appropriate when using a model with numerous outputs, from which a subset is requested to interact with variables in other coupled models. This component delivers the simulation results to the user.

Each time a pseudo-model is executed, the ISS is solicited to convert or translate the state variables (the output) of the pseudo-model to MIMIC state variables (the ones used in the interaction codes). Thus, they can be used as inputs for the other pseudo-models.

### The user’s layer or how the users communicate with MIMIC

Interactions in MIMIC are generated from the users’ instructions, covering the following 2 aspects: (a) the interaction code itself written by the user and so-called UIM (user interaction model) and (b) the control of the simulation, the so-called UC (user simulation control).

In both cases, the instructions are processed through the “User–MIMIC communication” component. Indeed, we want the kernel being untouched and safe from external process to guarantee the platform integrity.

In MIMIC, we consider the UIM and UC as pseudo-models. This offers numerous interests. It keeps the architecture consistent as a whole; the state variables of the interaction can be kept and made available easily; it gives the potential to build a hierarchical embedding of applications; the user can define active observers (acting as controllers operating according to results gained in the interaction code).

**Fig. 7.**
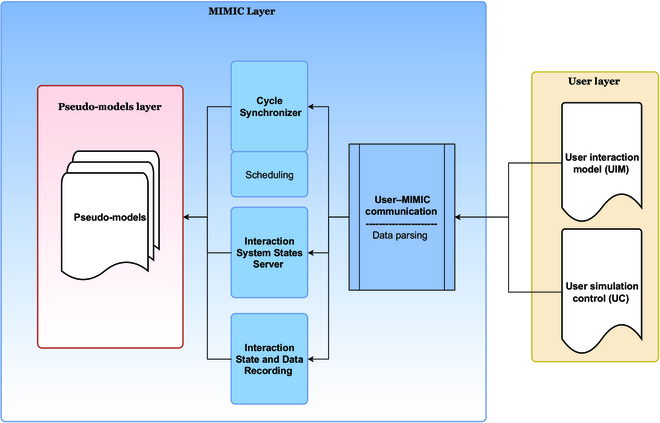
Structure of the interaction between the MIMIC (functioning presented in Fig. 4) and the users. The data provided by the user are read and used to create metadata for the construction of the interaction in the “User–MIMIC communication.” Then, the metadata is used in creating and filling the pseudo-models.

The user–MIMIC communication component is, in fact, a parser that creates metadata for building the pseudo-models and the connections to the kernel (Fig. 7).

**Fig. 8.**
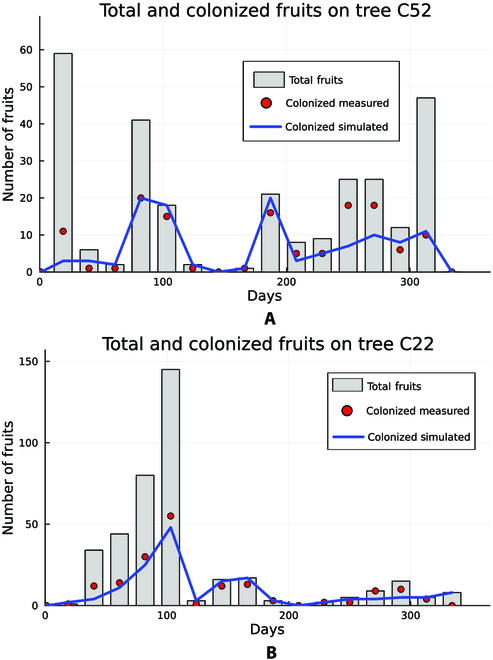
Simulation results compared to field data on 2 coffee trees in Indonesia. (A) stands for tree C52 and (B) stands for the tree C22. Points represent the recorded number of harvested fruits colonized by CBBs on 6 fruiting branches of the tree. The line represents the results of the simulation by MIMIC at the same date than the observation. Bars represent the total observed harvested fruits (healthy and colonized).

#### User interaction model (UIM)

Users write in Julia language, the code describing the interaction between the models, using the pseudo-model’s state variables and pseudo-model’s calls. However, using Julia to encode the UIM is not a requirement: (a) The interaction code can also be an “external” model, belonging to the model layer. This method leads to a higher level of complexity and lower performance since requesting to be wrapped in a pseudo-model. (b) Next, if the level of coupling is weak, operating only on the inputs and outputs of the models, forming a “shared coupling” as defined in [23], then the user is not requested to provide any UIM. The simulation starts directly from the initial schedule and runs from the explicit cycle input and output definitions described for the pseudo-model generation.

#### User simulation control (UC)

The UC defines the information related to the execution of models for simulation. These descriptions are presented in the form of a file in YAML format. The UC is used to parse the model metadata and create the variables to be mobilized during the interaction execution process. An example of such a file is given in Table 3. Some input fields are required, while others, left by the user, are filled with a default value. In the table, the name given to the model is “Tree.” However, if no value was provided, then “model_n,” where n is the rank of the model in the file,would have been the default value.

**Table 3.** YAML model definition pattern example on a plant model.

*Field name*	*Subfiled*	*Type of value*	*Value*
*Is model active*		Boolean	True
*Name*		String	Tree
*Version*		Any	a0.4
*Language*		String	R
*Path to File*		Path from root	D:/MyPrg/Plants/TreeSim.R
*Time data*	***Nature***	Keyword (*)	temperature
	***Unit step size***	Value	20
	***End simulation***	Value	2350
	***Start simulation***	Value	0
*Variables data*	***State variables***	Vector	[Yield, biomass]
	***Inputs***	Vector	[ RelativeTempe ]
	***Outputs***	Vector	[Yield]
*Interaction data*	***MyRefDir***	Path from root	/Fungus2Tree.jl
	***Language***	String	C

*Day, month, or temperature.

The model rank, i.e., the order in which the model is described, sets the model priority. The order of models is essential, because the models’ priority is based on their location in the list definition. This priority is considered when several model calls must be processed on the same event date.

### Executing MIMIC

MIMIC executes in a 2-step process. The first one, “MIMICinit,” runs the initialization of MIMIC and generates the pseudo-model codes and the initial schedule. The second one, “MIMICmain,” launches the schedule. MIMIC generates by default a CSV (comma-separated values) file containing the values of all variable states at each simulation step.

### Development and dissemination

This framework is primarily aimed at scientists and engineers in the agricultural and environmental sectors looking to estimate P&D effects on plants on long term to assess possible resilience of plants. We intend that this implementation be a user-friendly tool, with an open access code for further development. The modular structure of the architecture should provide the flexibility to adjust and refine the interactions. Finally, its parameterization is easy to understand and is adapted to users who are not specialists in software development.

The tool was developed in Julia, an open-source high-level dynamic programming language [52]. It offers the advantage of calling scripts written in other languages popular in the plant science modeling community (MATLAB, R, etc.), and Julia remains close to them in its syntax. This choice is also dictated (a) by the possibility to use existing codes (models) without direct rewriting; (b) by the performances, especially in terms of speed of the language; and (c) by a growing number of libraries produced by a growing community of contributors. This argument reflects concerns of many scientists in numerous fields [53].

In the user layer, translating variables from one model to another using variable manipulation with ISS or/and arithmetic operation is straightforward, even in Julia. The code is written in a file using a notepad, with a “.jl” extension or through a code editor. In the YAML file, the name of the functions and the path to the code are listed. The advantage of YAML over other file formats is its simplicity to be read. Writing instructions for this file type is simple and understandable for users of all professional backgrounds.

Access to the source code is free via GitHub: https://github.com/Houssem-Triki/MIMIC. The kernel code can be found there including the case study. The tool is coded as a package that can be downloaded using the Julia REPL command line. Users will find the templates for the YAML input files. These documents can be edited using notepad software or any code editor. The Git provides documentation on MIMIC and the case study example, for which a template sheet data file is provided for the fruiting data, allowing users to test different tree production and harvesting situations.

### Study case: Sumatra, Indonesia (data used for simulation)

The case study example involving the CBB, fructification, and human models described in the previous section enables us to illustrate a simple application mobilizing MIMIC and its performances.

The models (CBB, coffee, and human) are loaded into MIMIC using a parameter template such as in Table 3. The complete template for this case study can be found on MIMIC’s Git.

**Table 4.** Parameters used for interaction in the case study instancing the definition in Table 3.

*Field name*	*Subfiled*	*Value*
*Is model active*		*True*
*Name*		MIMIC
*Version*		0.9
*Language*		Julia
*Path to File*	*./MIMIC-main.jl*
*Time data*	***Nature***	*Day*
	***Unit step size***	1
	***End simulation***	355
	***Start simulation***	1
*Variables data*	***State variables***	[fruitsCohorts, Ag, Ar]
	***Inputs***	
	***Outputs***	[fruitsCohorts]
*Interaction data*	***MyRefDir***	*/Interaction.jl*
	***Language***	*Julia*

#### Parameters of the coupling

MIMIC’s parameters used in the study case are shown in Table 4. The step time of the simulation will be determined from the parameters that are entered into the time data field of MIMIC’s model parameters. For more details, each model and, thus, each pseudo-model are characterized by the simulation’s step time (in cycles), the simulation’s start time (start simulation), and the simulation’s end time.

Users need to specify the models’ state variables (CBB, coffee, and human) that are relevant to the interaction in MIMIC in the “variables data” field.

#### Results of the case study

The interaction between the coffee tree and the CBB models is constructed around the attraction of the pest on the type of fruits (from the CBB point of view). A specific detail concerns the way fruit maturity is considered in both human and fruit models.

Among the datasets, 2 trees were chosen (named C52 and C22), exhibiting dissimilar fruiting dynamics (Fig. 8, bars). Tree C52 produced ripe fruits for nearly the entire year, whereas tree C22 produced fruits for only the first one-third of the monitoring period.

The proportion of infested fruits among harvested fruits for the 2 trees is the result of a MIMIC simulation in which an initial number of CBB was introduced. MIMIC simulated the attack dynamic accurately.

There was one significant peak in tree C22 and 2 less significant ones. The models succeeded in their task of fitting both the primary peak and one of the minor peaks, both in terms of amplitude and length. There was a minor shift in the position of the final weaker peak.

As for tree C52, there were 3 major and 2 secondary peaks. Two of the major peaks and one of the secondary peaks were well fitted by the model in amplitude. The last major peak was underestimated in intensity. The first peak at the beginning of the monitoring did not correspond to the observations, which is due to the initial conditions. Dynamic systems may be sensitive to initial conditions, and a small change can result in a different outcome [54].

#### Performances and coding key figures

The MIMIC code is compact, with reduced complexity. The complexity is linear as a function of the number of events. Table 5 shows its performance in the study case. Most of the simulation time is spent on initialization, which consists of interacting with the user layer. The rest of the run time is split between the MIMIC’s kernel components and the external models.

Initialization clearly consumes the majority of simulation time. This is because Julia is executing a code for the first time. On the first run, Julia simulates a virtual machine to compile the algorithms. Hence,the second run requires less memory (3,720 GB with 28.9 s for the first run compared to a 727 MB with 3.81 s for the next one). 

**Table 5.**
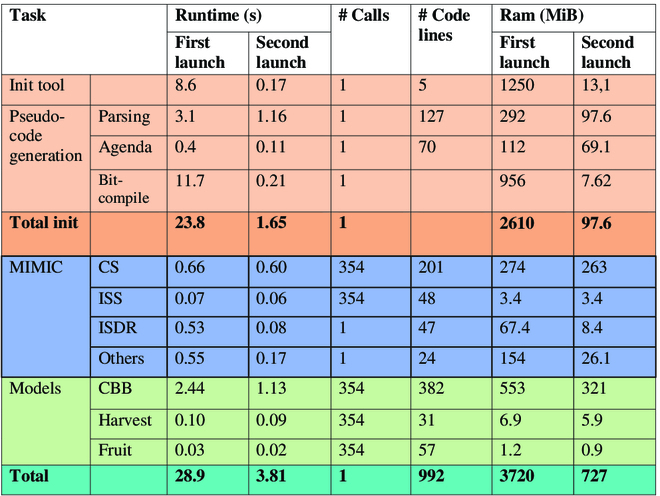
Study case runtime performance and code complexity on the study case. User model and initialization;MIMIC’s kernel; external models. The runtime and memory allocation columns contain information for a first and second execution (Intel Core i7-11850H @ 2.50 GHz, RAM 64 GB, Windows 10).

## Discussion and Perspectives

In this paper, we introduced a methodology and tool for linking models, specifically plant and P&D models. Here, we discuss the underlying assumptions of our proposal and present some near-term perspectives.

### Cohort assumption

Cohorts simplify the complexity of models and their associated data. Indeed, factorization makes it easier to manipulate the outputs/inputs of the various modeling formalisms to make them interact. Processes are no longer treated on an individual basis, but as homogenous sets with the same behaviors and parameters. Cohorts are commonly used in health sciences; their benefits are frequently addressed [55].

In our case, the usage of cohorts reduces the complexity of the interaction. Consequently, factoring into cohorts simplifies the handling of the outputs/inputs of multiple modeling formalisms during interaction, due to the unit of time that characterizes each cohort Assumedly, we suppose that the plant models organize organs by cohort [20]. This assumption cannot always be mobilized. However, in the case of FSPMs, factorizations can be performed post hoc by establishing cohorts clustering all organs of same physiological stages and appearance date (thus, sharing similar environmental conditions). Conversely, in crop models, the cohorts can also be defined subdividing the PBM organ compartments, gradually identifying the cohorts from the number of new organs appeared [56].

Similarly, on P&D models, the cohorts can be defined from population dynamics from development stages. This allows compatibility between the interacting models.

#### About spatialization

In our proposal, we assume spatialization as an implicit aspect of the models. This point will not be detailed here. However, in some cases, geometrical (spatialization) can be related to aging (for instance, distance from soil or distance from crown). MIMIC may then be used for coupling spatialized models with ones that are not, providing the fact that the UIM can explicit the request spatialized inputs from the model implicit output ones.

### Feedback and ISS

Pseudo-models’ output transformation is performed by the ISS component that allows the manipulation of the variables directly without having to go through modifications on the intervening models. This discharges the users to ensure complete compatibility between models’ outputs. It is up to them to handle the different levels of feedback of the interaction through the UIM. The users are given complete freedom on how to choose the principles of the interactions outside the models. This situation contrasts to other formalisms where inner modifications are requested, such as in DEVS [42], and where discrete time is driven by the models and events.

Despite DEVS formalism extensions, improvements, and adaptation to community needs [57], this problem is not solved including within the agronomy community [58], as illustrated in the Record project [59], an initiative stopped recently.

In MIMIC, the users can decide to make a shared coupling by not providing an UIM code, when the outputs between models are used as inputs. They only need to indicate these outputs/inputs in the UC file. Users can also explore different concepts on the interaction with the UIM code. They can make the interaction correspond to one of the other frameworks proposed by Siad et al. [28].

### Choosing Julia, user-friendly meta programming

Julia is a fast, dynamic, easy to use, and open-source programming language; it is accessible to the modeling community. Its syntax bears a resemblance to the common program languages used by modelers (Python, R, and MATLAB). In addition, the UIM does not need to be written as a complex algorithm, as shown on our example.

When using dynamic programming (by requesting user–MIMIC parser to produce the pseudo-models), Julia’s speed is advantageous because MIMIC’s input (UC and UIM) is preprocessed and an optimized code is generated. The parser in MIMIC will take most of the time and resources at the first run of the program. However, after it, the simulations are swift (Table 5) and users can update the simulation parameters without losing performance.

The interaction complexity can be analyzed according to the number of models involved in the system and, more precisely, to the number of operations handled by the ISS, which translates variable sets between the pseudo-models and the kernel. Ignoring the initialization stage (that can be considered as a constant value plus a linear cost in terms of the number of models involved), the complexity related to the requested model exchanges is drastically limited in MIMIC compared to an embedded approach. With MIMIC, the number of set translations leads to a linear complexity with the number of models involved, because for n models, n translations are required. In the embedded case of an upfront coupling between each model and the other, the complexity is defined by the number of models mutually coupled. For n models, there are potentially n-1 variable set translations (to connect to the other models), which is, to say, up to n^*^(n-1) translations. 

### Perspectives

It is advantageous to have a modular structure for MIMIC when integrating additional components and adding extra capabilities. Additionally, it is designed to be compatible with other platforms, such as OpenAlea, even if not implemented yet [33]. We do not have a specific constraint on the type or nature of the involved models (except their ability to run in a stop and go way, and potentially input and output variables that can be expressed as cohort variables). Thus, the proposed approach is versatile and generic, which increases the coupling options.

We plan to design a generic plant model implementation within MIMIC. Users would then simply need to set plant-specific parameters and incorporate their other models (e.g., P&D).

## Conclusion

The literature on the limitations of model coupling (plants and P&D) reveals that feedback is not taken into consideration and that it is challenging to find an appropriate framework without modifying the structures and logic of the models involved.

In this paper, the proposed framework was developed to address this issue and to provide a platform to couple prebuilt models. This framework is depicted by the tool MIMIC.

MIMIC provides the ability to couple models in a variety of ways, ranging from direct data transmission between models to more complex interaction principles that require a third-party tool to add a new element and modify the outputs of the interacting models. MIMIC’s primary benefit is that it gives users the tools and freedom to construct their own interactions.

Given that all the coupled models and components of MIMIC are represented as pseudo-models, the implementation with Julia’s high-speed metaprogramming enables rapid interaction outcomes.

The implementation of MIMIC in Julia provides a flexible and straightforward algorithmic environment for users with limited coding experience and a good trade-off with other modeling-oriented programming languages (R, Python, MATLAB, etc.).

The case study presented in this paper illustrates the MIMIC framework, coupling a coffee tree fructification model and a CBB model.

As a result, the proposed approach emphasizes the challenge on coupling plant growth and P&Ds interactions’ models.

## Data Availability

The data and source code that support the findings of this study are openly available at https://github.com/Houssem-Triki/MIMIC.
